# Supplementary material for: Gastrula‐Premarked Posterior Enhancer Primes Posterior Tissue Development Through Cross‐Talk with TGF‐β Signaling Pathway
Source: Adv Sci (Weinh). 2025 Jun 29;12(36):e00895. doi: 10.1002/advs.202500895 (PMC12463030; doi:10.1002/advs.202500895)
Supplement: Supplementary file 1 — Supporting Information [file ADVS-12-e00895-s006.pdf]

ADVANCED SCIENCE
Open Access

Supporting Information

for *Adv. Sci.*, DOI 10.1002/advs.202500895

Gastrula-Premarked Posterior Enhancer Primes Posterior Tissue Development Through Cross-Talk with TGF-β Signaling Pathway

Yingying Chen, Fengxiang Tan, Qing Fang, Lin Zhang, Jiaoyang Liao, Penglei Shen, Yun Qian, Mingzhu Wen, Rui Song, Yonggao Fu, He Jax Xu, Ran Wang, Cheng Li, Zhen Shao*, Jinsong Li*, Naihe Jing* and Xianfa Yang**

Supporting Information

for Adv. Sci., DOI: 10.1002/advs.202500895

Gastrula-premarked Posterior Enhancer Primes Posterior Tissue Development through Cross-talk with TGF-β Signaling Pathway

Yingying Chen[1,8], Fengxiang Tan[1,8], Qing Fang [2,8], Lin Zhang[3,8], Jiaoyang Liao[4], Penglei Shen[1], Yun Qian[3], Mingzhu Wen[1], Rui Song[1], Yonggao Fu[1], He Jax Xu[3], Ran Wang[5], Cheng Li[2,6,]*, Zhen Shao[7,]*, Jinsong Li[3,]*, Naihe Jing[1, 9,]*, and Xianfa Yang[1,] *.

*Corresponding author. Email: cheng_li@pku.edu.cn (C.L.), shaozhen@sinh.ac.cn (Z.S.), jsli@sibcb.ac.cn (J.L.), jing_naihe@gzlab.ac.cn (N.J.), yang_xianfa@gzlab.ac.cn (X.Y.).

This PDF file includes:

Supplementary Figures 1-10
Supplementary Table 1-6

Figure S1. screening and identification of posterior development related pre-marked distal regulatory elements

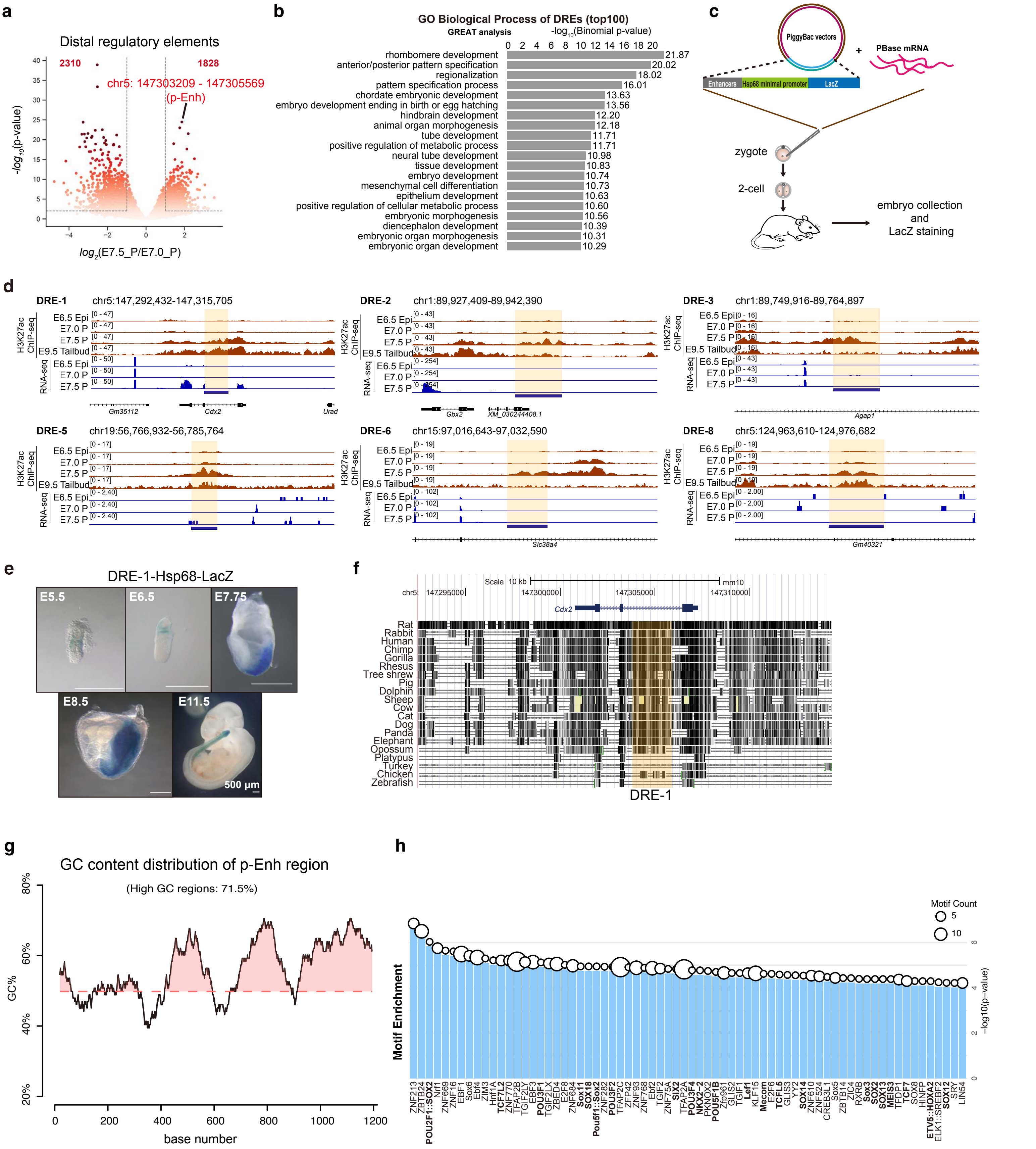

Figure S1. Screening and identification of posterior development related pre-marked distal regulatory elements.

(a) Volcano plot illustrating the posterior-specific distal regulatory elements with significant changes in activity. p-values are calculated by using a modified two-sided t-test (two-sided) in MAnorm2 (see Statistical analysis).

(b) GO biological process enrichment analysis (GREAT) of top-100 DREs, sorted by $-\log_{10}$ (Binomial p-value). Analysis performed using default parameters with a whole-genome background model. Detailed information regarding the top-100 DREs is provided in Table S1, Supporting Information.

(c) The diagram illustrating the experiment details about the transgenic embryo screening system for analyzing the activity of DREs during embryonic development.

(d) IGV snapshots of H3K27ac ChIP-seq signals of indicating DREs.

(e) DRE-1-Hsp68-LacZ transgenic embryos illustrating robust activity of p-Enh throughout continuous developmental stages. Scale bar: 500 μm.

(f) UCSC browser snapshot showing high conservation of DRE-1 across various species.

(g) GC content distribution of p-Enh region. Genomic regions with over 50% GC are highlighted in red.

(h) Ranked list of 70 transcription factor (TF) consensus motifs identified by FIMO[95] and Motifscan[96] in the p-Enh genomic region. Identified TF motifs are ranked by $-\log_{10}(p\text{-value})$, from FIMO analysis.

Figure S2. Expression patterns of p-Enh-derived transcripts

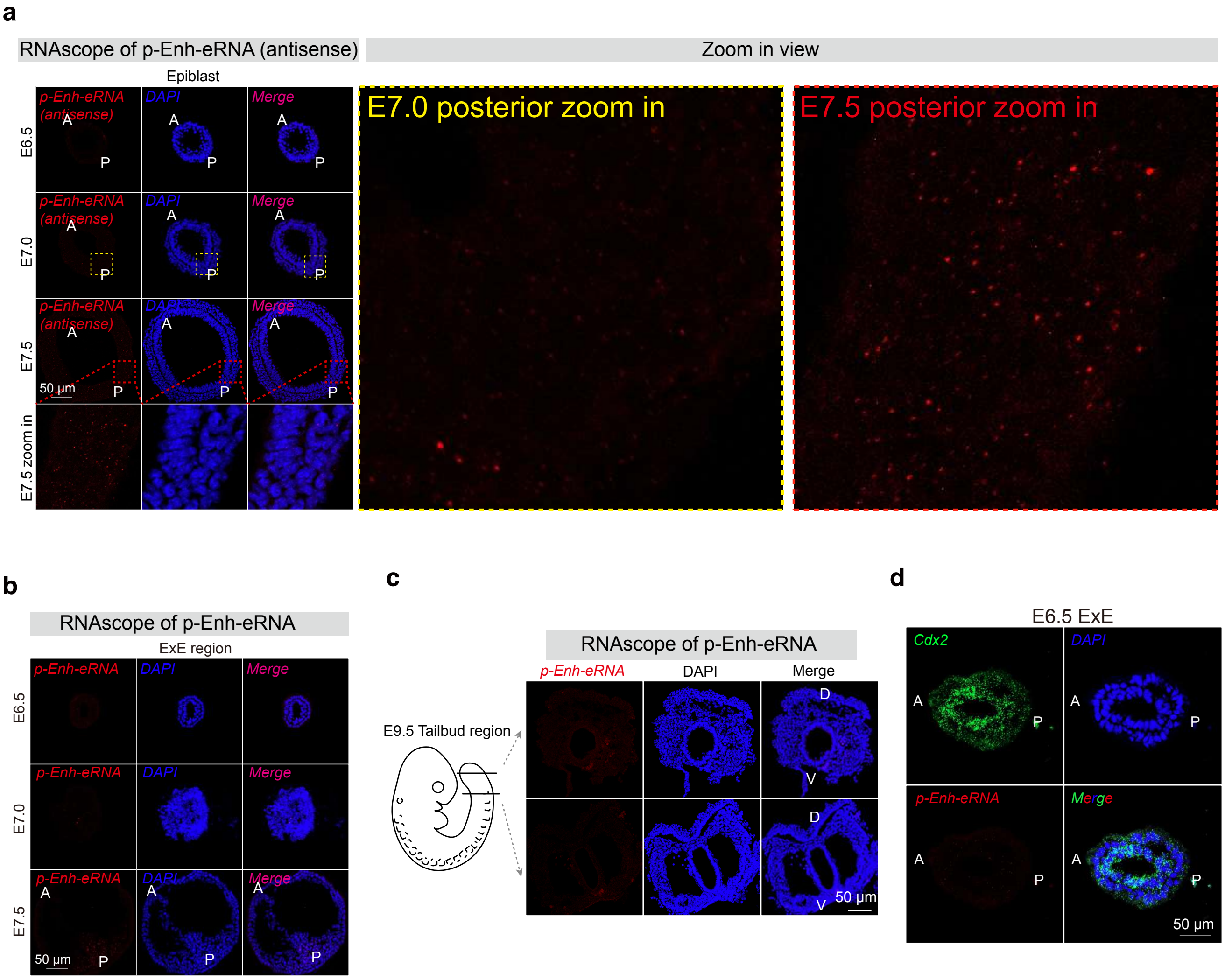

Figure S2. Expression patterns of p-Enh-derived transcripts.

(a) RNAscope results of p-Enh-eRNA (antisense) in the embryonic regions from E6.5 to E7.5. Results of zoom in view on the right reporting the presence of antisense transcripts in the E7.5 posterior region. A: anterior region, P: posterior region. Scale bar: 50 µm.

(b) RNAscope results targeting p-Enh-eRNA in E9.5 tailbud regions. D: dorsal region, V: ventral region. Scale bar: 50 µm.

(c) RNAscope results of p-Enh-eRNA in ExE regions from E6.5 to E7.5. A: anterior region, P: posterior region. Scale bar: 50 µm.

(d) Co-staining of *Cdx2* mRNA and p-Enh-eRNA using RNAscope in the E6.5 ExE regions.
Scale bar: 50 µm.

Figure S3. p-Enh-KO mouse embryos exhibit embryonic lethality

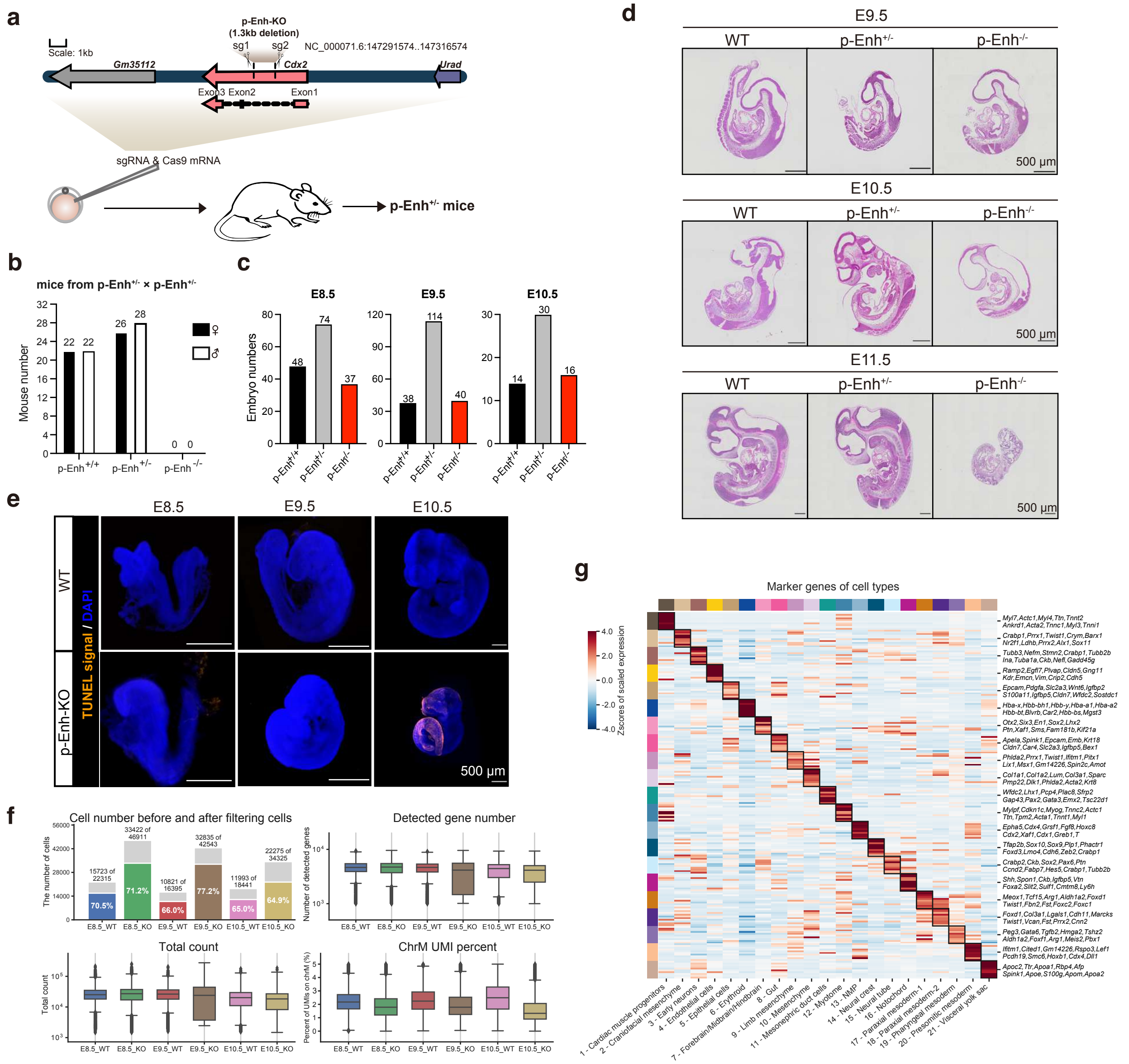

Figure S3. p-Enh-KO mouse embryos exhibit embryonic lethality.

(a) The diagram illustrating the strategy for generating p-Enh-KO embryos. Two sgRNA sets targeting boundaries of p-Enh locus were shown.

(b) Genotypes of offspring from self-crossing of p-Enh$^{+/-}$ mice.

(c) Genotypes of embryos at different developmental stages from self-crossing of p-Enh$^{+/-}$ mice.

(d) HE staining results of embryos with different genotypes at different developmental stages. Scale bar: 500 μm.

(e) Lightsheet imaging of whole-mount TUNEL results of WT and p-Enh-KO embryos at different development stages. Scale bar: 500 μm.

(f) Quality control of single-cell RNA-seq data.

(g) Heatmap showing top-10 marker genes of each cluster. The complete list of marker genes for all clusters can be found in Table S3, Supporting Information.

Figure S4. Developmental deficiency in p-Enh-KO embryos

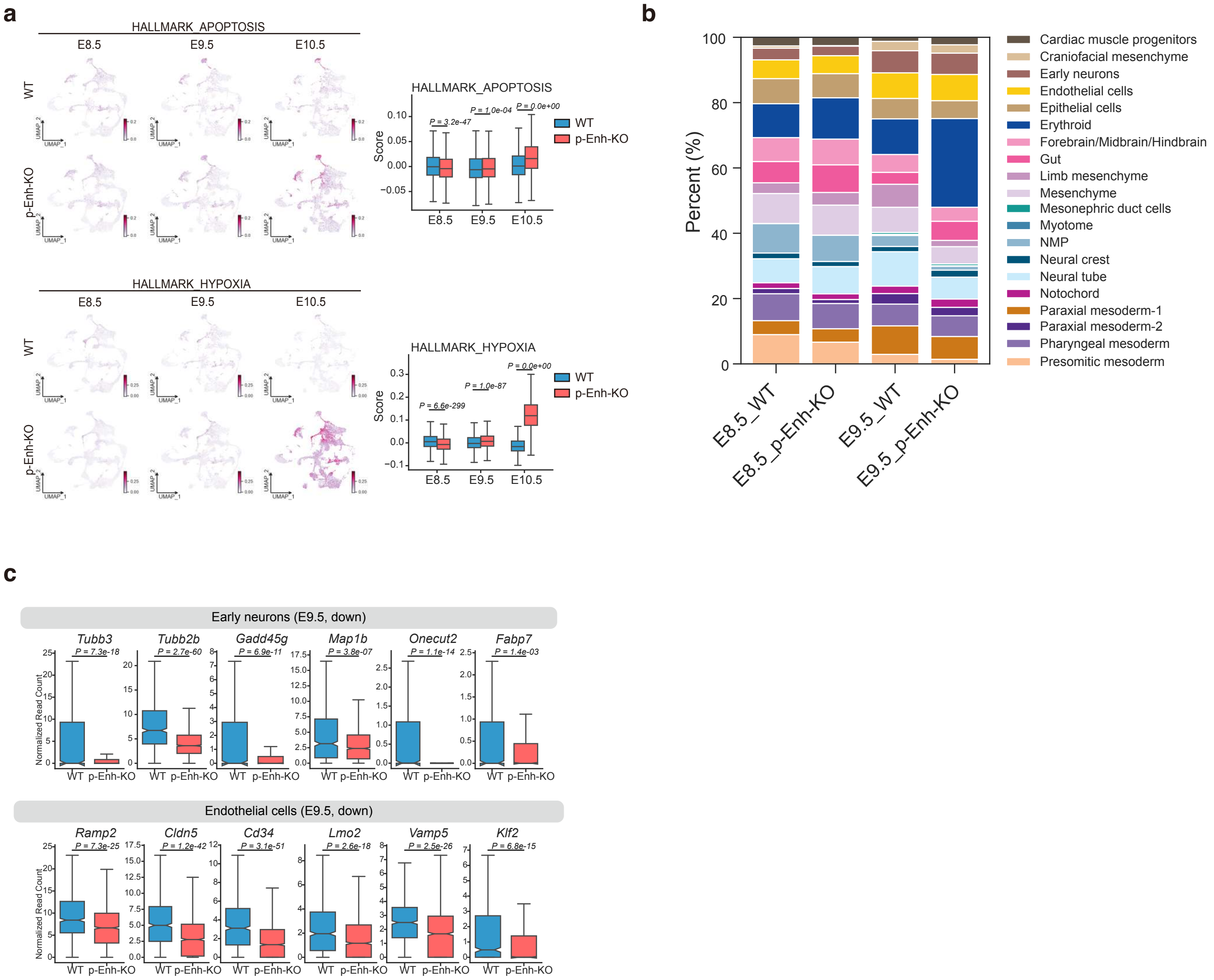

Figure S4. Developmental deficiency in p-Enh-KO embryos.

(a) Activities of apoptosis and hypoxia signaling were assigned based on hallmark gene list from GSEA dataset. Boxplot illustrating the corresponding activity score in each sample. The p-values are calculated using Wilcoxon rank sum test (two-sided) via the FindMarkers function in Seurat.

(b) Percentage of cell abundance of different clusters in each sample.

(c) Boxplot illustrating normalized read count of marker genes with significant expression changes between WT and p-Enh-KO embryos in indicated clusters. p-values are calculated by using a two-sided Wilcoxon rank sum test in Seurat FindMarkers Function.

Figure S5. Reconstruction of digital A-P axis

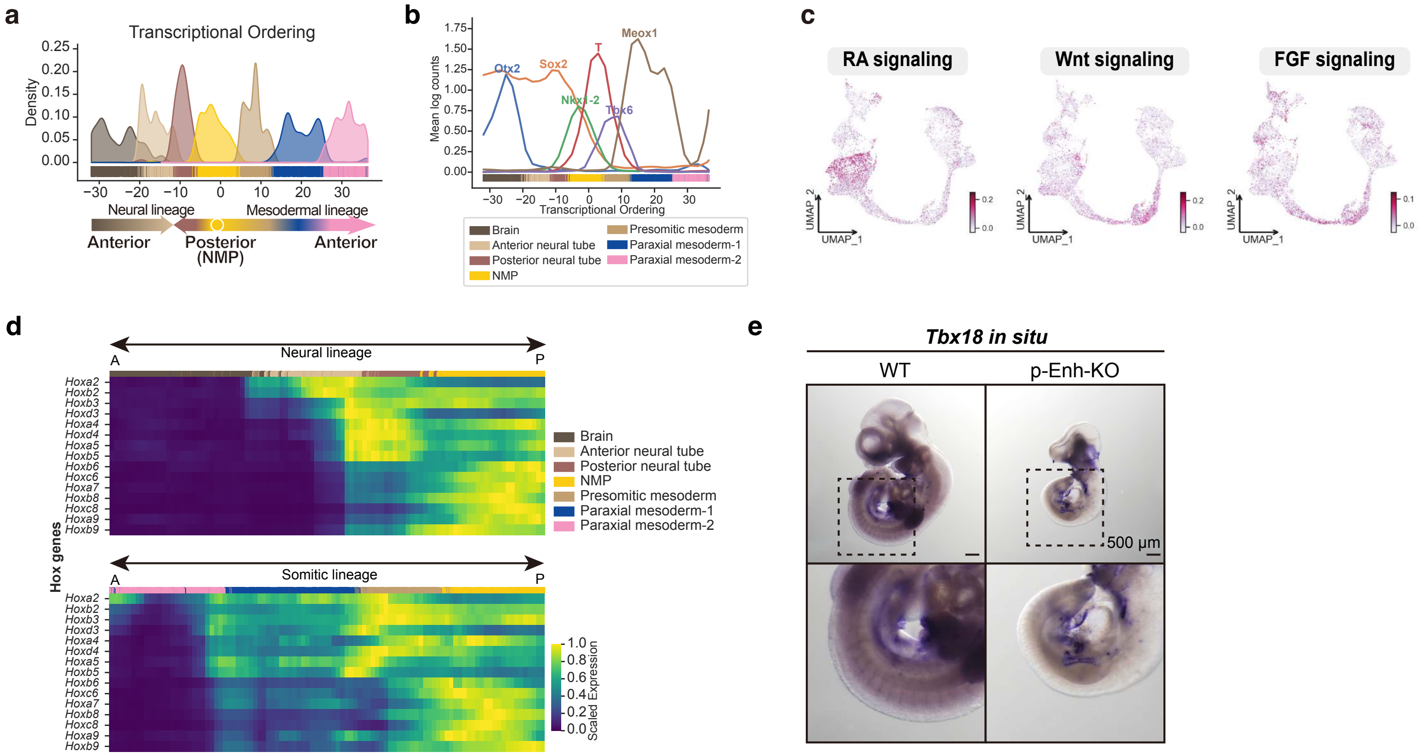

Figure S5. Reconstruction of digital A-P axis.

(a) Cell density distribution along the transcriptional ordering. Two different origins of neural tube cells are indicated based on RNA velocity result in Figure. 3a.

(b) The expression patterns of selected transcription factors along the one-dimensional A-P axis.

(c) RA, Wnt, and FGF signaling activities projected along the reconstructed A-P axis UMAP. Signaling activities are measured through gene sets (MM15186, MM3864, MM5178) from GSEA datasets[124]. The signaling enrichment scores are calculated based on AddModuleScore function in Seurat package.

(d) Heatmap showing the expression patterns of *Hox* gene family along the one-dimensional transcriptional ordered A-P axis. Scaled expression is calculated based on the output of the NormalizeData function from the Seurat package, and further normalized to a [0, 1] range per gene using min-max scaling.

(e) *In situ* hybridization for *Tbx18* in E10.5 WT and p-Enh-KO embryos, with enlarged views highlighting the signals around somite structures. Scale bar: 500 μm.

Figure S6. No detectable phenotypes of brain, branchial arch, and heart in p-Enh-KO embryos

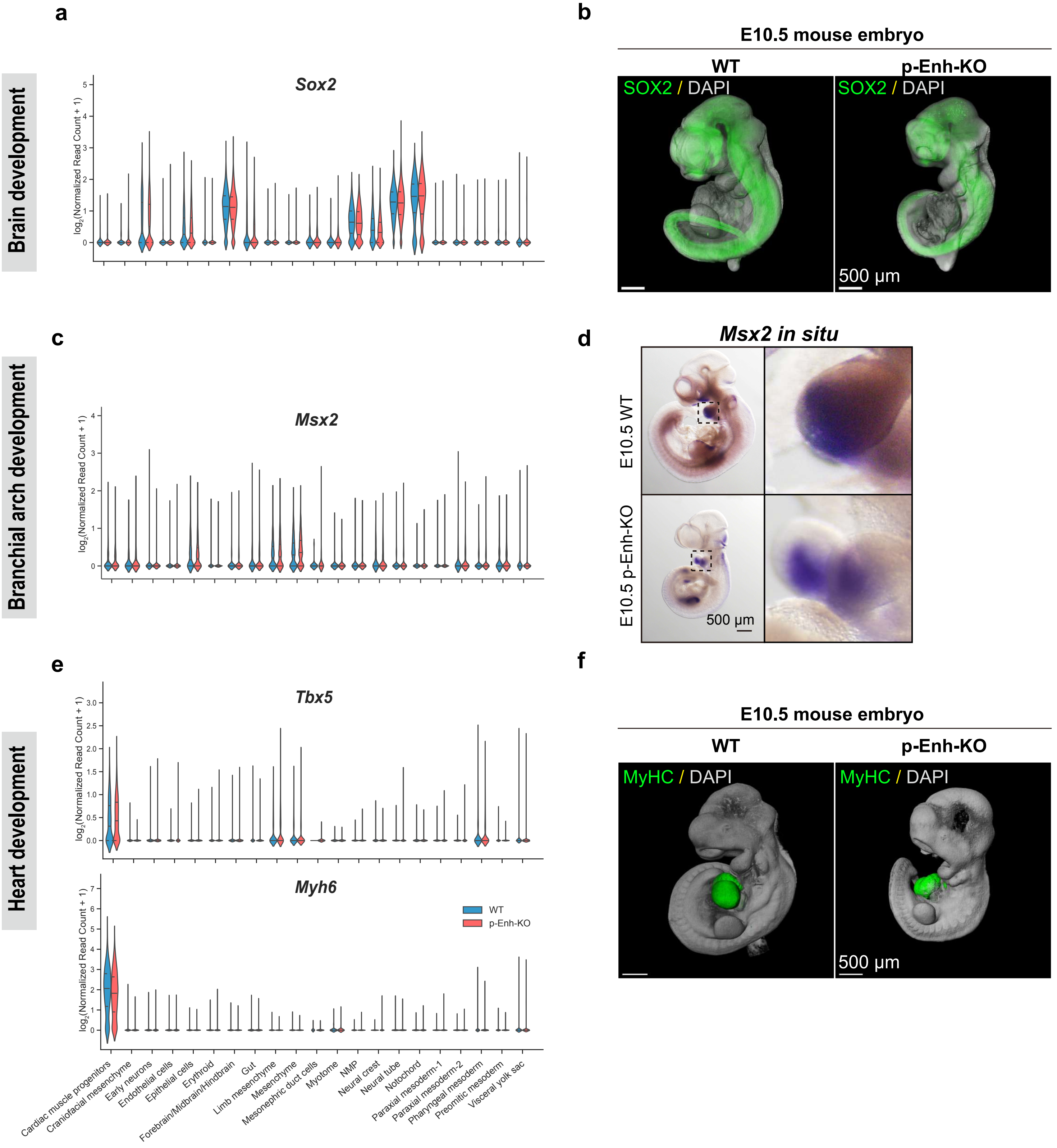

Figure S6. No detectable phenotypes of brain, branchial arch, and heart in p-Enh-KO embryos.

(a) Expression profiles of *Sox2* in WT and p-Enh-KO embryos from single-cell transcriptomic data.

(b) Whole-mount immunofluorescence for SOX2 in WT and p-Enh-KO mouse embryos.

(c) Expression profiles of *Msx2* in WT and p-Enh-KO embryos from single-cell transcriptomic data.

(d) *In situ* hybridization for *Msx2* in E10.5 WT and p-Enh-KO embryos, with enlarged views highlighting signals around the branchial arch region. Scale bar: 500 μm.

(e) Expression profiles of *Tbx5* and *Myh6* in WT and p-Enh-KO embryos from single-cell transcriptomic data.

(f) Light-sheet imaging showing whole-mount immunofluorescence for MyHC in E10.5 WT and p-Enh-KO embryos. Scale bar: 500 μm.

Figure S7. Generation of p-Enh-KO and Cdx2-KO mESC and their differentiation deficiency

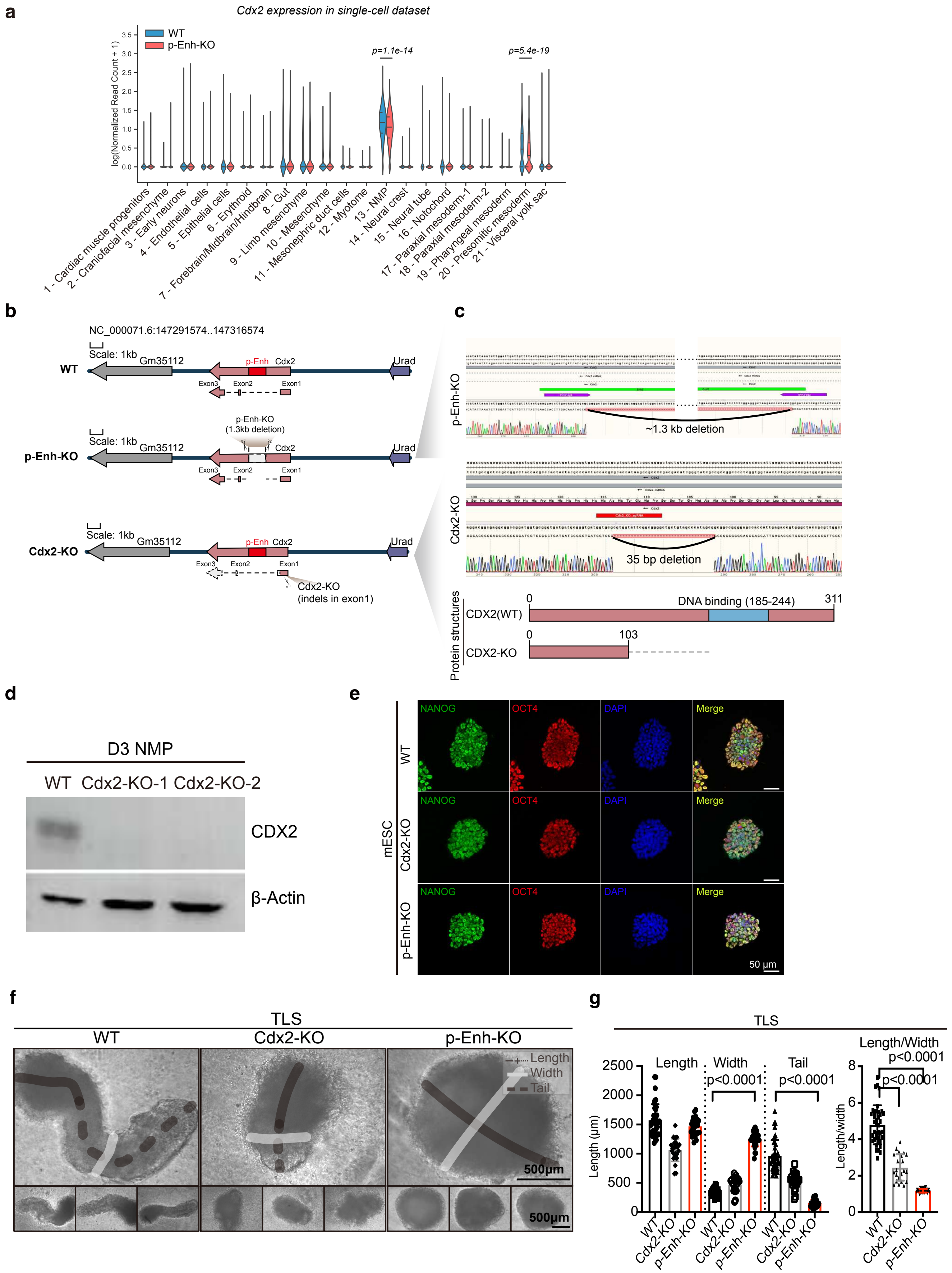

Figure S7. Generation of p-Enh-KO and Cdx2-KO mESC and their differentiation deficiency.

(a) The expression pattern of *Cdx2* in different cell clusters in single-cell dataset of WT and p-Enh-KO embryos. p-values are calculated by using a two-sided Wilcoxon rank sum test in Seurat FindMarkers Function.

(b) Diagram illustrating the strategy for generating p-Enh-KO and Cdx2-KO mESC.

(c) Sanger sequencing results showing successfully knock out of p-Enh-KO and induced indel in Cdx2-KO cell lines. The protein structure diagram showing the absence of CDX2 DNA binding domain in Cdx2-KO cellline.

(d) Western blotting result for CDX2 protein in NMP cells.

(e) Immunofluorescence results of store cultured mESC of WT, Cdx2-KO and p-Enh-KO cell lines. Scale bar: 50 μm.

(f) Bright field images of TLS with distinct genotypes.

(g) Statistical analysis of morphology parameters of TLS in each group. Statistical significance is determined by one-way ANOVA.

Figure S8. Transcriptome analysis revealed distinct functions between p-Enh and *Cdx2*

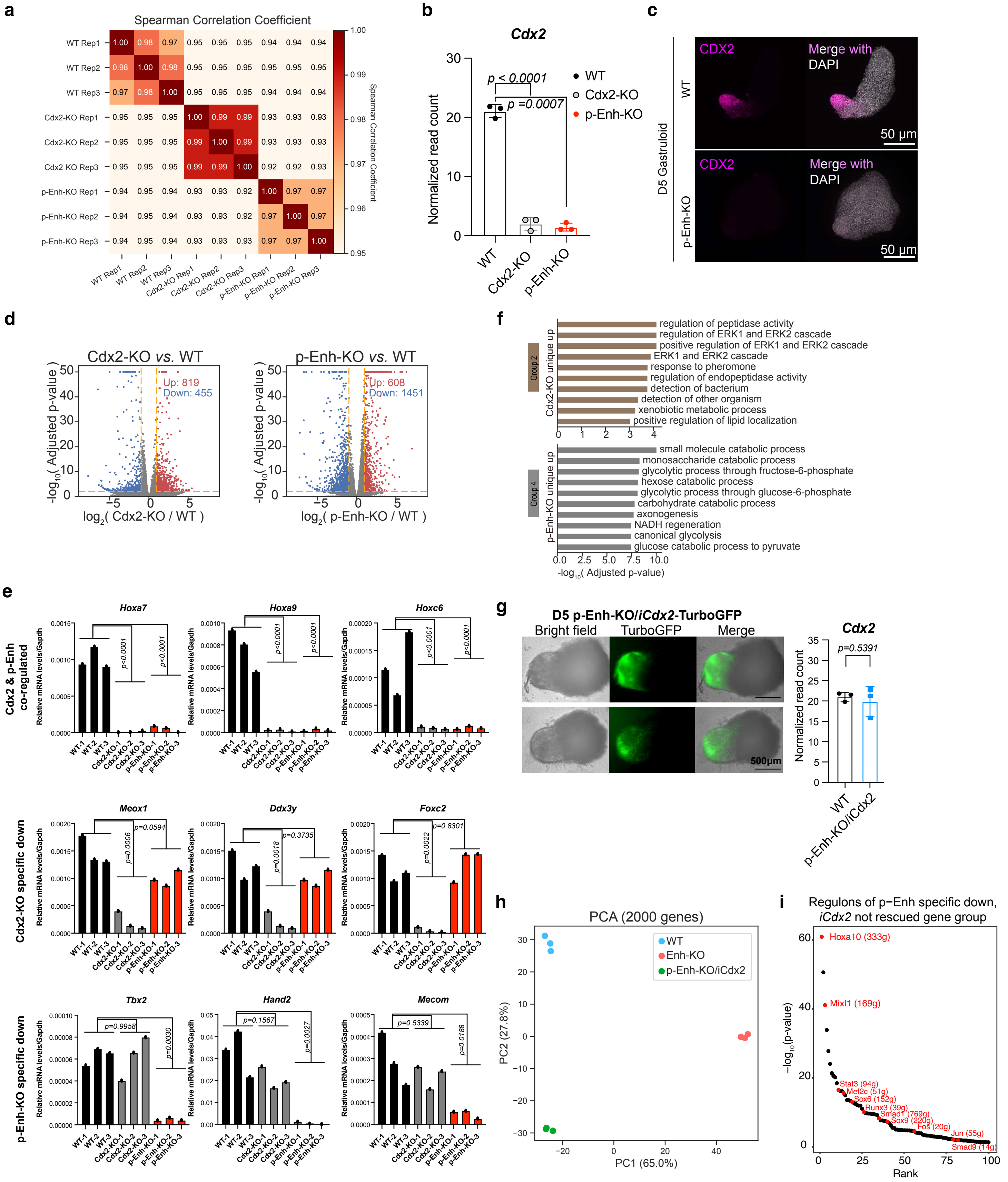

Figure S8. Transcriptome analysis revealed distinct functions between p-Enh and *Cdx2*.

(a) Heatmap showing spearman correlation coefficient among different transcriptome data from WT, Cdx2-KO and p-Enh-KO groups.

(b) Normalized read count of *Cdx2* in different gastruloid samples.

(c) iDISCO immunofluorescence results targeting CDX2 protein in D5 gastruloids from WT and p-Enh-KO groups. Scale bar: 50 μm.

(d) Differentially expressed genes (DEGs) (p-value < 0.01, fold change >2) of Cdx2-KO vs. WT, and p-Enh-KO vs. WT. The up-regulated genes are labeled in red and the down-regulated genes labeled in blue. p-values are calculated by using a Wald test (two-tailed) in DESeq2 (see Statistical analysis).

(e) qPCR validation of gene expression in D5 Gastruloids for *Cdx2* & p-Enh co-regulated genes, Cdx2-KO specific down genes, and p-Enh-KO specific down genes. Each genotype includes three independent biological replicates (n=3). Statistically significant differences between groups are determined by one-way ANOVA.

(f) Gene Ontology (BP) results of Group 2 and Group 4 genes defined from Figure. 4h. p-values are calculated using hypergeometric test (one-sided) and adjusted for multiple testing using BH method.

(g) Bright field image and TurboGFP expression in D5 p-Enh-KO/iCdx2 gastruloids. Normalized read count of *Cdx2* transcripts confirm successful overexpression. p-value is calculated using unpaired t-test.

(h) Principal component analysis result of gastruloid transcriptomes from WT, p-Enh-KO, and p-Enh-KO/iCdx2 groups.

(i) Regulons of p-Enh-KO specific down, *iCdx2* could not rescued gene groups (Group 5-B). Regulons overlapped with TGF-β signaling pathway are highlighted in red.

Figure S9. Loss of p-Enh leads to genome-wide epigenomic remodeling

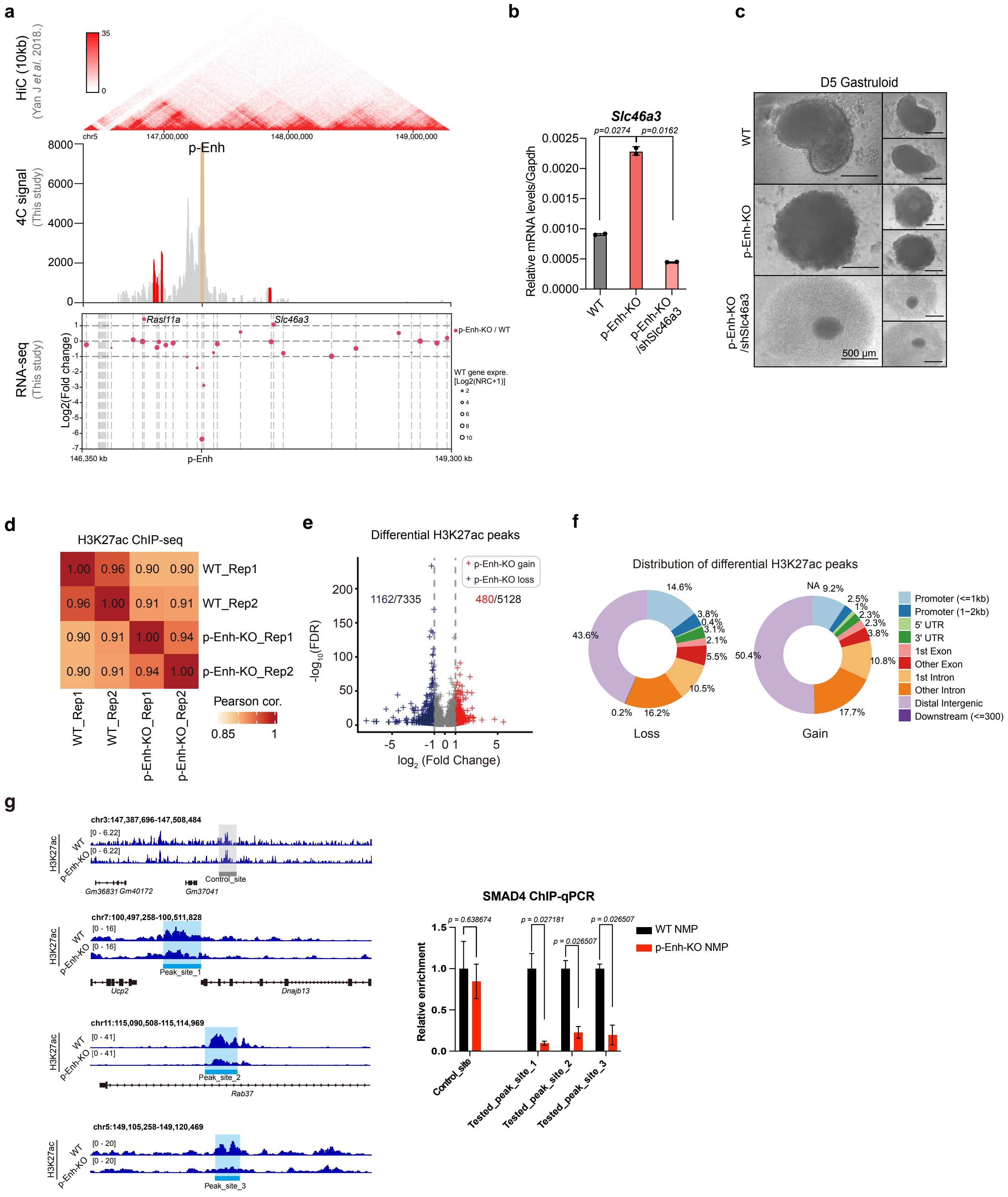

Figure S9. Loss of p-Enh leads to genome-wide epigenomic remodeling.

(a) Visual inspection of coverage profiles near the p-Enh viewpoint reveals chromatin interactions overlap with TADs identified by Hi-C[66] and correspond to gene expression in this region (chr5: 146,350,000-149,300,000). Top: Hi-C map of mESCs at 10-kb resolution. Middle: 4C-seq profiles using p-Enh as the viewpoint (three replicates); significantly interacting regions are highlighted in red. Bottom: RNA-seq results for all genes within this chromatin region. Genes with fold change > 2 are considered significantly differentially expressed.

(b) qPCR results showing knockdown efficiency of Slc46a3 using shRNA. Statistical significance is determined by unpaired t-tests.

(c) Bright-field images showing D5 gastruloid in each group. Scale bar: 500 µm.

(d) Heatmap showing Pearson Correlation Coefficient among H3K27ac ChIP-seq data from WT and p-Enh-KO NMPs.

(e) Volcano plot illustrating differential H3K27ac peaks. Significant differential peaks are defined with FDR <0.05 and log2(Fold Change)>=1.

(f) Genomic distribution of p-Enh-KO lost and gained peaks.

(g) IGV snapshots of representative sites showing decreased H3K27ac signals in p-Enh-KO NMPs compared with WT group, alongside the control locus with unchanged H3K27ac levels. SMAD4 ChIP-qPCR in WT and p-Enh-KO NMP cells targeting peak sites in these sites. p-values are calculated using unpaired t-test.

Figure S10. Involvement of TGF-β signaling in p-Enh's functions and the transcriptome analysis

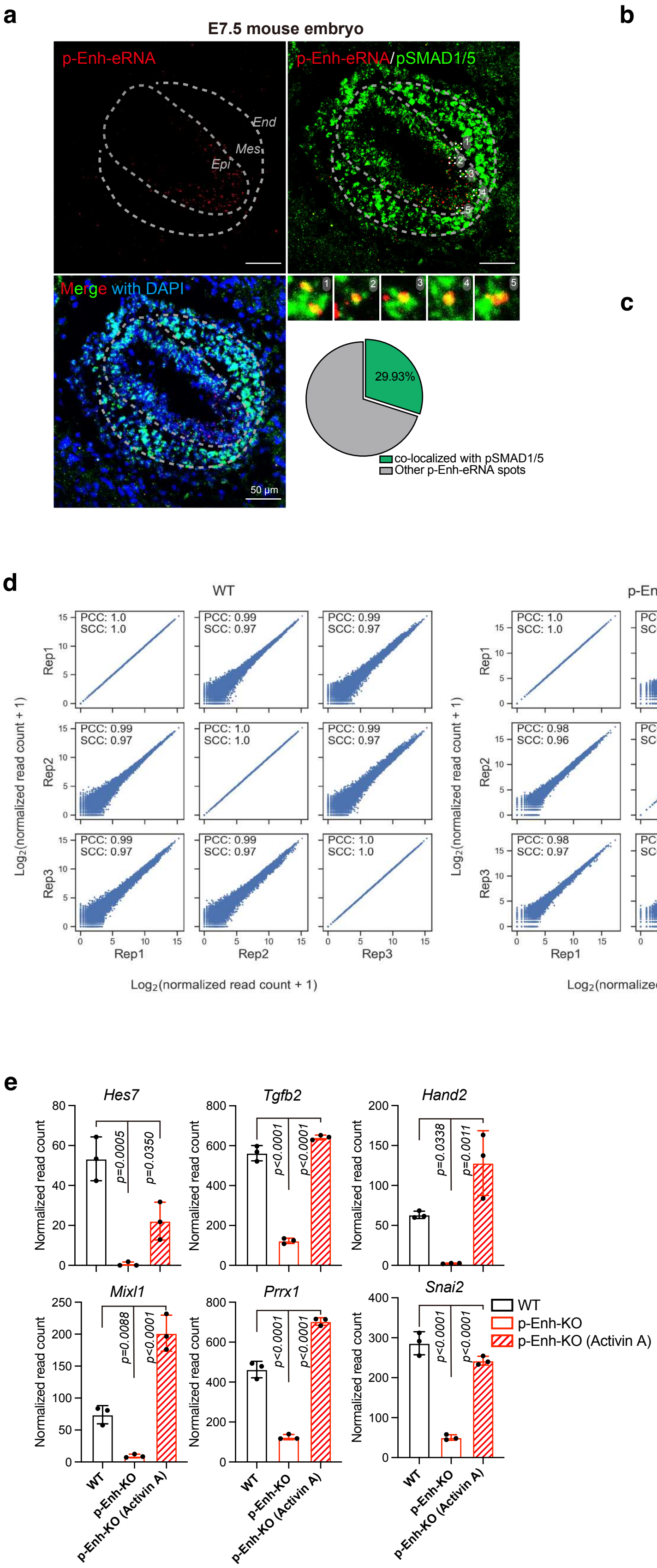

Figure S10. Involvement of TGF-ß signaling in p-Enh's functions and the transcriptome analysis.

(a) Co-staining of pSMAD1/5 protein and p-Enh-eRNA in E7.5 mouse embryos. Selected co-localization sites are highlighted with zoom-in views. Scale bar: 50 μm. Quantification of co-localization is performed using ComDet v0.0.5.

(b) pZW1-snoVector system used for over-expression of p-Enh-eRNA in p-Enh-KO mESC. Scale bar: 100 μm.

(c) Bright field images of D5 gastruloids from WT and p-Enh-KO groups in SB431542 treatment (10 μM) condition. Scale bar: 200 μm.

(d) Scatter plots showing correlation among transcriptome data from WT, p-Enh-KO, and p-Enh-KO (Activin A) groups.

(e) Expression pattern of example genes in WT, p-Enh-KO and p-Enh-KO (Activin A) gastruloids. Statistical significance is determined by one-way ANOVA.
